# A case of a hemodialysis patient with secondary hyperparathyroidism who was resistant to etelcalcetide treatment but not to cinacalcet hydrochloride

**DOI:** 10.1007/s13730-021-00664-0

**Published:** 2021-11-17

**Authors:** Hironori Nakamura, Masanori Tokumoto, Mariko Anayama, Shigekazu Kurihara, Yasushi Makino, Katsuhiko Tamura, Masaki Nagasawa

**Affiliations:** 1 Department of Nephrology, Shinonoi General Hospital, 666-1 Ai Shinonoi, Nagano, 388-8004 Japan; 2 Department of Nephrology, Japanese Red Cross Fukuoka Hospital, Fukuoka, Japan

**Keywords:** Ca-sensing receptor, Calcimimetics, Etelcalcetide resistance, Secondary hyperparathyroidism

## Abstract

Although both cinacalcet and etelcalcetide are calcimimetics that directly inhibit parathyroid hormone (PTH) secretion by activating the calcium (Ca)-sensing receptor (CaSR), their binding sites are different. We report a first case of a hemodialysis (HD) patient with secondary hyperparathyroidism (SHPT), in whom cinacalcet, but not etelcalcetide, could reduce serum intact PTH (i-PTH) levels. A HD patient received total parathyroidectomy (PTx) with auto-transplantation 16 years earlier. Due to SHPT relapse, cinacalcet was started at 7 years after PTx. His i-PTH levels had been controlled with both 75–100 mg of cinacalcet and 4.5 μg/week of calcitriol for a year before switching from cinacalcet to etelcalcetide. At 1 month following the switch, his serum i-PTH level increased to 716 pg/mL. The dose of etelcalcetide was gradually increased and finally reached the maximal dose of 45 mg/week. Because even the maximal dose of etelcalcetide for > 4 months did not reduce his serum i-PTH levels to < 700 pg/mL, etelcalcetide was switched to 50 mg/day of cinacalcet, which reduced the levels to 208 pg/mL at 2 months after the switch. Genomic sequencing test using whole blood revealed no mutation in the portion including Cys 482 of CaSR gene. The patient was resistant to etelcalcetide treatment but not to cinacalcet, suggesting the possibility that the enlarged parathyroid gland has some change in the portion including Cys 482 in the CaSR gene. Therefore, considering the possibility of etelcalcetide resistance during SHPT treatment should be kept in mind.

## Introduction

Etelcalcetide is a newly developed, intravenous calcimimetic that is effective in reducing parathyroid hormone (PTH) levels [1]. It directly inhibits the PTH secretion by activating the calcium (Ca)-sensing receptor (CaSR), but its binding site is different from that of cinacalcet. Although there are some proportion of patients who could be considered resistant or non-responders to etelcalcetide after the switch from cinacalcet, to our knowledge, precise information on individual cases has not been released. Hence, we report the first case of a hemodialysis (HD) patient with secondary hyperparathyroidism (SHPT) who was resistant to etelcalcetide treatment but not to cinacalcet.

## Case report

**Fig. 1:**
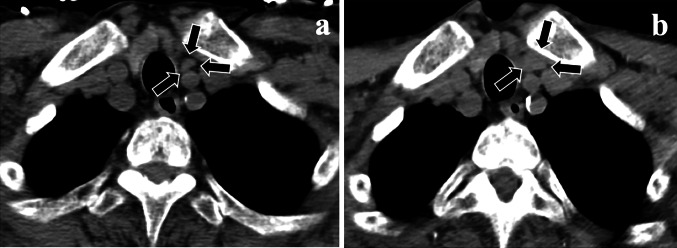
Demonstrable images of computed tomography showing the change in the size of an enlarged parathyroid gland in the neck, over 9 years. Foot note: **a** Arrows show an enlarged left lower parathyroid gland measuring 7.6 × 9.9 × 24.5 mm (volume: 1.30 mL) in the neck in 20XX-6. **b** Arrows show the same enlarged parathyroid gland measuring 11.0 × 18.6 × 28.0 mm (2.83 mL) in 20XX + 3

**Fig. 2:**
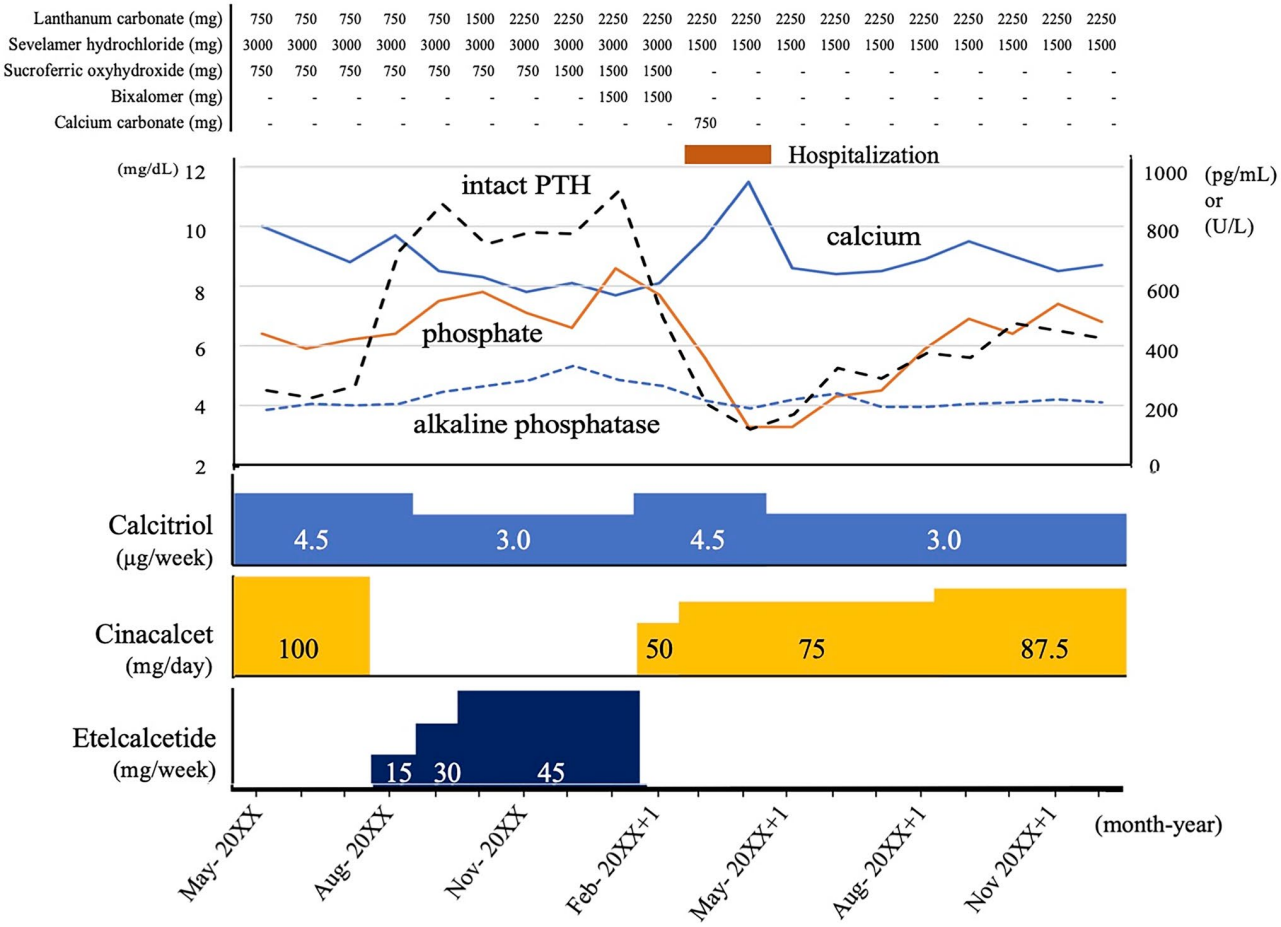
The monthly changes in the serum levels of calcium (mg/dL), phosphate (mg/dL), alkaline phosphatase (U/L) and intact parathyroid hormone (pg/mL); daily dose of cinacalcet (mg) and P binders (mg); and weekly dose of calcitriol (μg) and etelcalcetide (mg) from May 20XX to December 20XX + 1 including 6 months of etelcalcetide treatment. The brown bar between March and April 20XX + 1 indicates his hospitalization due to lacunar infarction

A 29-year-old man started receiving peritoneal dialysis in 20XX-26 due to chronic glomerulonephritis; his renal replacement therapy was switched to HD in 20XX-20. He gradually developed SHPT, and his intact PTH (i-PTH) levels reached 770 pg/mL with enlarged parathyroid glands under the treatment of 30 μg/week of maxacalcitol in 20XX-16. Therefore, he received total parathyroidectomy (PTx) with auto-transplantation to his right forearm. Four parathyroid glands were resected, and all the glands demonstrated nodular hyperplasia (top right, 38 × 18 × 15 mm; bottom right, 17 × 10 × 8 mm; top left, 11 × 9 × 8 mm; and bottom left, 15 × 10 × 8 mm). After PTx, his serum i-PTH levels decreased to 30 pg/mL. Following SHPT relapse, 25 mg of cinacalcet was initiated in 20XX-9, and gradually increased up to 100 mg by 20XX-6. In 20XX-6, an enlarged parathyroid gland measuring 7.6 × 9.9 × 24.5 mm [volume 1.30 mL, calculated using the 3D image analysis (Synapse Vincent, Fujifilm, Co.,Tokyo, Japan)] was detected in the neck (shown in Fig. 1a) by computed tomography (CT), and an enlarged auto-transplanted parathyroid gland measuring 11.2 × 9.7 × 6.1 mm was detected on echogram. After 20XX-6, SHPT was treated with 75–100 mg/day of cinacalcet and either 2.0–4.5 µg/week of calcitriol or 15 μg/week of maxacalcitol. His serum i-PTH concentration was controlled at a level of < 300 pg/mL for a year until July 20XX. During this period, lanthanum carbonate, sevelamer hydrochloride, and sucroferric oxyhydroxide were prescribed as phosphate (P) binders at a dose of 750, 3000, and 750 mg, respectively. Cinacalcet was switched to etelcalcetide for better compliance in August 20XX. As a result of the initiation of etelcalcetide at the starting dose of 15 mg/week, his serum i-PTH levels increased to 716 pg/mL after a month. The dose of etelcalcetide was gradually increased and finally reached the maximal dose of 45 mg/week. However, this treatment did not reduce the patient’s serum i-PTH levels, and hence the doses of lanthanum carbonate and sucroferric oxyhydroxide were increased up to 2250 and 1500 mg, respectively, and 1500 mg of bixalomer was also added. In February 20XX + 1, etelcalcetide was switched back to 50 mg/day of cinacalcet, because etelcalcetide was not effective. After 2 months, his serum i-PTH levels decreased to 208 pg/mL. As his serum P levels reduced by this improvement in serum i-PTH levels and the P-restricted diet administered during hospitalization due to lacunar brain infarction for 2 months from March to April 20XX + 1 reduced his serum P levels, the dose of sevelamer hydrochloride was reduced, sucroferric oxyhydroxide and bixalomer were withdrawn, and 750 mg of Ca carbonate was initiated instead during his hospitalization. Thereafter, the dose of cinacalcet dose was increased up to 87.5 mg according to the elevation of serum i-PTH levels. Figure 2 shows the monthly dose of each P binder, the monthly changes in the serum levels of Ca, P, alkaline phosphatase, and i-PTH, the daily dose of cinacalcet, and the weekly dose of calcitriol and etelcalcetide during the 20 months (from May 20XX to December 20XX + 1) including the 24 weeks of etelcalcetide treatment. In 20XX + 3, a CT examination revealed an enlarged parathyroid tissue measuring 11.0 × 18.6 × 28.0 mm in size (volume 2.83 mL) in the left lower lobe (shown in Fig. 1b). Meanwhile, the enlarged auto-transplanted parathyroid gland regressed to 10.9 × 4.0 × 3.6 mm in size. SHPT is being currently treated with 4.5 μg/week of calcitriol and 7 mg of evocalcet, which is a novel calcimimetic developed to decrease the gastro-intestinal adverse effects of cinacalcet [2]. 7 mg of evocalcet is equivalent to 87.5 mg of cinacalcet in terms of the suppression of PTH secretion. The i-PTH gradient, calculated using the i-PTH concentration (342 pg/mL) in the auto-transplanted side of the forearm after 5 min avascularization divided by the i-PTH concentration (373 pg/mL) in the other side (arteriovenous fistula side) without avascularization, was 0.92, suggesting that the remaining parathyroid gland in the neck is mainly secreting PTH. Genomic sequencing test using whole blood revealed no mutation in the portion including Cys 482 of CaSR gene (BEX CO., LTD., Tokyo, Japan).

## Discussion

A randomized double-blind, double-dummy active clinical trial demonstrated that etelcalcetide was not inferior to cinacalcet in reducing the serum PTH concentrations [1]. In that study, the median of weekly etelcalcetide dose was 15.0 mg, and the median of daily cinacalcet dose was 51.4 mg during the efficacy assessment phase. According to the result, an estimated equivalent dose of etelcalcetide for 100 mg/day of cinacalcet is considered to be 30 mg/week. In the present case, because even a maximal dose of etelcalcetide could not suppress excess i-PTH secretion, and the patient’s serum i-PTH level decreased to the target level in 2 months after conversion to cinacalcet, we concluded that the patient was resistant to etelcalcetide treatment. As shown in Fig. 2, compared with cinacalcet treatment, lower serum Ca level, and lower dose of calcitriol were observed during the etelcalcetide treatment, because increased serum PTH level due to the switch from cinacalcet to etelcalcetide induced elevation of Ca–P products. As Ca and vitamin D decrease the serum i-PTH levels [3, 4], these factors could partially affect the increase in i-PTH levels during etelcalcetide treatment even in the present case. However, when we compared serum i-PTH levels between etelcalcetide and cinacalcet treatments at the similar levels of Ca and P with the same dose of administrated calcitriol, the serum iPTH level was higher in etelcalcetide treatment. Briefly, on September 20XX, during 30 mg/week of etelcalcetide treatment, the serum levels of i-PTH, Ca, and P were 872 pg/mL, 8.5 mg/dL and 7.5 mg/dL, respectively. On November 20XX + 1, during 87.5 mg of cinacalcet treatment, the serum levels of iPTH, Ca, and P were 452 pg/mL, 8.5 mg/dL and 7.4 mg/dL, respectively. These findings suggest that serum i-PTH level was effectively suppressed on cinacalcet use, compared with etelcalcetide use, when we took into account the study result that cinacalcet 50 mg/day was equivalent to etelcalcetide 15 mg/week for inhibition of serum PTH level [1]. We could not demonstrate the precise mechanism, because the conservative treatment has been continued without parathyroidectomy of the remaining parathyroid glands. Therefore, there is a lack of information regarding the *CaSR* gene of the causal parathyroid gland; however, we assume that the difference in the binding site between cinacalcet and etelcalcetide could have induced the different efficacy in controlling PTH levels. Both cinacalcet and etelcalcetide inhibit PTH secretion by activating the CaSR, which has extracellular, transmembrane, and intracellular domains. Cinacalcet binds to the transmembrane region of the CaSR. Meanwhile, etelcalcetide binds to Cys 482 on the outer membrane, which is critical to the pharmacological activity of CaSR, and functions as a CaSR agonist. The formation of the covalent disulfide bond between the Cys 482 on the human CaSR and the cysteine residue of etelcalcetide is critical for the pharmacological activity of the peptide, and the extent of its interaction correlates with the activity, leading to the rapid activation of CaSR [5, 6]. In the present case, genetic analysis revealed that no systemic abnormalities in the *CaSR* gene of the site to which etelcalcetide should bind; however, we think that a mutation occurred locally in the parathyroid gland as the parathyroid gland hyperplasia progressed.

The other possibilities are considered as follows. Although there is a report for the up-regulating effect of cinacalcet on CaSR [7], there is no report regarding up-regulation of VDR expression by cinacalcet. However, other calcimimetic compounds, such as R568 and AMG641, have been reported to increase CaSR and VDR expression in uremic hyperplastic parathyroid glands. For example, treatment for 2 weeks with AMG641, a calcimimetic compound more potent than cinacalcet, upregulated the expression of both CaSR and VDR in rats with renal insufficiency to the similar levels of control [8]. Similarly, treatment of 5/6 nephrectomized rats with AMG416 (etelcalcetide) for 6 weeks increased the expression of CaSR two-fold compared to the vehicle. The expression levels after treatment with AMG416 were similar to those observed in normal rats [9]. Although each calcimimetic compound has been suggested to increase CaSR and VDR in parathyroid cells, there is no direct comparative studies between the two compounds. Therefore, further studies are needed to determine if the two compounds have different effects on the expression of CaSR and VDR.

A previous report suggested that a specific *CaSR* single nucleotide polymorphisms (SNPs) was associated with variation in PTH response to cinacalcet [10]. However, to the best of our knowledge, the relationship between *CaSR* SNPs and the response of PTH to etelcalcetide has not been reported. Among those *CaSR* SNPs, if some SNPs suppress PTH secretion in response to cinacalcet treatment but not to etelcalcetide treatment, the SNPs might be involved in the higher PTH levels during etelcalcetide treatment as observed in the present case. At present, it is difficult to conclude whether the less inhibitory effect of etelcalcetide on PTH secretion can be simply explained by *CaSR* SNPs. The examination for interaction between *CaSR* variants and response to etelcalcetide in patients with SHPT might provide further explanations about responsive differences between the two drugs.

We believe that the relapse of SHPT can be attributed to an enlarged parathyroid gland in the neck because of the following reasons: first, regression of the enlarged auto-transplanted parathyroid gland was observed; second, a parathyroid gland in the neck was enlarged; and third, the i-PTH gradient was 0.92, suggesting that the remaining parathyroid gland in the neck is mainly secreting PTH [11].

In conclusion, the finding that cinacalcet, but not etelcalcetide, effectively suppressed PTH secretion in this patient with relapsed SHPT suggests the possibility that the enlarged parathyroid gland has some change in the portion including Cys 482 in the CaSR gene. Although further investigations, including genetic examination in the parathyroid glands, are required to clarify the precise mechanism, considering the possibility of resistance to etelcalcetide treatment in patients with SHPT is important at least.
